# Information and shared decision-making are top patients' priorities

**DOI:** 10.1186/1472-6963-6-21

**Published:** 2006-02-28

**Authors:** Ami Schattner, Alexander Bronstein, Navah Jellin

**Affiliations:** 1 Department of Medicine, Addenbrooke's Hospital, School of Clinical Medicine, Cambridge University, UK; 2 Department of Medicine, Kaplan Medical Centre, Rehovot; Hebrew University Hadassah Medical School, Jerusalem, Israel

## Abstract

**Background:**

The profound changes in medical care and the recent stress on a patient-centered approach mandate evaluation of current patient priorities.

**Methods:**

Hospitalized and ambulatory patients at an academic medical center in central Israel were investigated. Consecutive patients (n = 274) indicated their first and second priority for a change or improvement in their medical care out of a mixed shortlist of 6 issues, 3 related to patient-physician relationship (being better informed and taking part in decisions; being seen by the same doctor each time; a longer consultation time) and 3 issues related to the organizational aspect of care (easier access to specialists/hospital; shorter queue for tests; less charges for drugs).

**Results:**

Getting more information from the physician and taking part in decisions was the most desirable patient choice, selected by 27.4% as their first priority. The next choices – access and queue – also relate to more patient autonomy and control over that of managed care regulations. Patients studied were least interested in continuity of care, consultation time or cost of drugs. Demographic or clinical variables were not significantly related to patients' choices.

**Conclusion:**

Beyond its many benefits, being informed by their doctor and shared decision making is a top patient priority.

## Background

Medical care has undergone profound changes in recent years. One of the most important changes with far reaching implications has been the advent of patient-centered medicine [1]. In contrast with the former paternalistic approach and the concentration on the medical/scientific model of disease, a novel attitude had been developed and promoted, redefining the physician's role. The new patient-centered approach emphasizing the patient's autonomy, values and preferences [2,3] was suggested to be highly beneficial [4-6]. However, it may be less widely accepted and applied in the field than is currently appreciated [7,8]. Another significant change involves the increasing impact of health maintenance organizations (HMO's). Restrictions imposed by the HMO's increasingly affect both physician's decisions and patients' access to health care [9-12]. The patient's reaction to all these changes has been infrequently studied. Current patient's priorities for improving their medical care were determined in this study.

## Methods

Six issues representing either themes central to the patient-physician relationship or to major administrative problems impacting patient care (3 each) were selected after a review of the literature and discussion among colleagues. The literature review covered articles in the English language published between 1994 and 2004 and indexed in PubMed on health care quality, access and evaluation; patient satisfaction/preferences and health services administration – patient-centered care. The designation of the selected issues was confirmed by a pilot study in which each item was correctly classified as pertaining to either medical or administrative aspects of care by ≥95% of 40 patients. These patients were randomly recruited from the population under care at the Kaplan Medical Centre – an academic hospital in central Israel. They formed a heterogeneous group representative of the population served by our center. Patients' motivation to improve elements in their medical care was then assessed. Asked if they were willing to participate in a study that examined their wishes to change and amend their medical care, all patients (100%) responded in the affirmative. When asked to indicate if they were 'very keen' to suggest changes, 'keen' or 'not interested', 34 patients were very keen (85%) and only 1 (2.5%) answered that he was not interested. Finally, patients were asked to indicate against each of the six items if they felt the issue was relevant to their own personal experience, and if relevant – if they considered it a common problem or a rare one. Responses were graded on a 5 point visual scale. All issues were judged both relevant *and *common by a large majority of the patients.

For the main study, hospitalized or ambulatory patients treated at our academic hospital, were approached consecutively and asked to participate. Both clinical settings were included in order to determine whether the experience of hospital admission affected patients' views or not. The point in time during the hospitalization or clinic visit varied for each patient, but this was not expected to have an effect on patients' responses since they were clearly asked for judgment of their overall experience of care and not of the current circumstances. The study was done during the spring of 2004. The patients received a brief verbal explanation saying only that their opinion was important; that declining would have nothing to do with their future care and participation was entirely voluntary and anonymous; and that rather than being interested in their immediate situation, we would like them to express their *general *opinion and preference. Consenting patients were handed a short form and asked to read it, consider, and mark their replies or indicate them to the researcher who marked it himself. The form was then collected. All patients currently treated in hospital or on ambulatory basis were eligible. Patients with cognitive impairment, language barrier (very few patients who spoke only Amhari, the language of Ethiopia) or those who were acutely ill and uncomfortable were excluded. The form [**see **Additional file 1] included 3 parts. In the *first*, the patient was asked as follows: if you could improve ONE thing about your medical care, what would you like to *change *as your FIRST priority or, what would you like the most to be *different*. Then, six options were provided, as follows – that you would have easier **access **to specialist physicians or the hospital; that the waiting time (**queue**) for tests would be shorter; that medications would **cost less**; that you would always be treated by the **same physician**; that the physician will **devote more time **to you; and that the physician will **explain **to you all about your illness and its possible treatment and will let you decide together. The first 3 options all relate to administrative problems. They are beyond the physician's jurisdiction and are not directly related to the patient-physician relationship. The last 3 options deal with the continuity of care, consultation time and the patient's information and autonomy. Thus they relate directly to medical care as administered by the physician.

The *second *part of the form included basic demographic and clinical details. On the *third part*, the patient was asked to make one additional selection, reflecting his or her second priority choice of the six issues [**see **Additional file 1]. To avoid bias related to an item's place on the list, 6 different types of forms were prepared. The study was approved by our Institutional Review Board. Chi-square and T test were used for the statistical analyses.

## Results

### The patients

Two hundred and seventy six patients were asked to participate and none declined. The response rate was therefore maximal and the same both before and after the verbal explanation. Two patients had selected more than one 'first priority' and were therefore excluded leaving 274 patients in the study. Patients' characteristics are shown in Table 1. These respondent characteristics are entirely representative of the full patient population at the study's hospital site. Patients' ages varied between 18 and 92 years-old (mean 54.4 ± 15.6, median 55 years-old). Their age distribution is given in the Figure.

### Their choices

Patients most frequently selected information and increased autonomy as their *most *desirable change, i.e. that physicians would provide them with full information about their illness and treatment and involve them in decisions. This option was chosen by 75/274 patients (27.4%) as their first priority and an *additional *36 patients wished for more autonomy as their second choice (Table 2). Altogether, 111 patients wished they had more information from their physicians and more participation in decisions as their first or second priority (111/548, 20.2%). Easier access to more sophisticated medical services or a shorter queue for tests were the next most wanted improvements (18% and 16%, respectively). Continuity of care came next (Table 2). Interestingly, the time their physician could spare for them or the cost of medications, were least likely to be among patient's chief concerns.

Since all patients were given the opportunity to choose their first priority for a change in their medical care as well as a second preference, we deemed it worthwhile to identify the most popular 'couplets' of choices. All combinations that were selected by >5% of the patients are presented in Table 3. Patient's information/autonomy, and smooth access/shorter queue to specialists and tests frequently appear among the 5 (out of 30 possible) combinations selected by ≥6% of the patients studied.

### Subgroup analysis

When patients' choices were examined in relation to their demographic variables (age group, gender, country of origin) or clinical variables (being hospitalized or in ambulatory care and main diagnosis) – no statistically significant relationship was found with any particular choice or a combination of choices (not shown). Education alone was partly related to patient's choices: patients who had elementary education only (n = 33), as opposed to those with high school or university education, showed significant preference for the three 'administrative' choices (Chi-square = 6.03, P = 0.01).

## Discussion

The adoption of the patient centered approach and the increasing role of managed care regulations have practically transformed medical care in recent years [3,13]. Paradoxically, patients' wishes and preferences have often remained unclear in this new setting. To determine which aspect of several key features of medical care (either 'organizational' or related to the patient-physician relationship) concern patients the most, we asked patients receiving care at an academic medical center for their opinion, using a clear short questionnaire.

Patients were very keen to participate, as the pilot study and the 100% response rate reveal. The heterogeneity of our study group (Table 1 and Figure) adds significance to our results, as does the fact that preferences were not found to vary with age-group, gender, income, education or illness-related characteristics. Patients with low level of education made more 'administrative' choices, but no particular selection was found to vary with education. In one study from California, patients' preferences for information and control were associated with being female, white, younger, more educated and having a higher income [14]. Our patients' selections however, were independent of demographic variables.

We found that patient's first priority for a change in a shortlist of 6 issues [**see **Additional file 1] was not related to the organizational aspect of care but to the patient-physician relationship. More than one fourth of the patients participating in the study selected getting more information from the doctor and taking part in decisions as their first priority for a change and improvement (Table 2). Unlike studies that addressed preferences of special patient populations and subgroups such as cancer patients or patients at the end of life, most patients reported here had no major decisions ahead. Nevertheless, many wished to become *partners *to the doctor's information and decision-making relevant to their care. In the case of some particular patient's subgroups however, alternative preferences might be found. For example, a prominent physician who became ill with cancer gave a moving account of being flooded with information yet he had a strong wish for a physician with authority who would make the 'best' decision on his behalf [15]. Such possible dichotomy between wanting greater information but not necessarily greater decisional control was also identified in several studies of cancer patients [16-18].

This abovementioned result may indicate not only a current deficiency in patient's preferred and attained levels of involvement, but also their needs for more autonomy and control over their care. Information and decisional control are multidimensional concepts and it can be argued that by choosing this item, participants have in fact selected two things. However, it is hard to separate the two. Not only are they both essential components of patient autonomy, but there can be no shared decision-making by the patient without the provision of information [19,20] including the existence of alternatives and their merits and shortcomings. The preference of our patients is in line with other observations. It has already been noted in several studies that although the patient centered model is widely advocated, its actual use in practice is probably rather limited [4,7,21-23]. *Patients' needs for information and a patient centered approach often remain unfulfilled*, as our study indicates and other studies support [4,24-28]. This is regrettable since besides complying with patients' wishes [5], adopting a patient-centered approach is associated with *several important health advantages *briefly presented in Table 4[5,29-38].

Although physicians' time has become a major concern [39], interestingly, patients were least concerned with the short amount of time that the physician could spare for them according to other studies [11,22]. The often disrupted continuity of care was also not a major issue for most. In a study that examined continuity alone, it was rated highly by patients – who were unwilling however to sacrifice a little more time or money to maintain it [40]. Continuity of care was rated more highly by doctors than by patients [24], suggesting that it can be partly related to the paternalistic model [41]. Only about 11–14% of the patients selected time or continuity as their first priority for a change, about a half or less of those who desired more information and participation in decisions (27.4%). Whether patients' first or second selection is examined, they identify access to specialists/hospital and waiting time for tests as important concerns, second only to patients' information/autonomy (Table 2). Although selected as first priority by just 16–18% of the patients, these issues keep appearing in patients' most favored combinations (Table 3), reinforcing their importance to the patients. At least one of them (access to specialists/hospital) can also be viewed as a measure of a desire for more patient control, rejecting the doctor's role as gatekeeper, imposed by the HMO's [12]. An inverse relationship between the intensity of managed care and patient satisfaction has already been noted in at least one study [42]. The cost of medications was much less important to most patients (<13%). Thus, a simple questionnaire may be utilized to learn not only of patient's priorities, but also of the possible existence of administrative interferences that hinder patient satisfaction with their medical care and need attention.

Our study had several limitations in that the issue selection was partially judgmental, the sample was not very large and the possible role of cultural reasons which may affect patients' selections was not explored. Also, patients' choices necessarily reflect their perceived problems with health services as administered locally. On the other hand, the issues presented for the patient's choice were based on a careful review of the literature (see under 'Methods') as well as a pilot study. The 100% participation rate of patients approached is a significant strength of the study. The study population was as heterogeneous as the population in central Israel, and may well be considered representative of the general Israeli population. Generalizability to other societies remains an open question. Since culturally, our population is essentially similar to other western countries, it is likely – but not proven – that our results may be generalized to other societies. This important point however, remains to be examined in future comparative international studies. Indeed, similar methodology may be easily adapted to study patients' priorities and wishes for improved medical care in any other location.

## Conclusion

Understanding the patient's narrative [43-45], providing all information [46] and joint decision-making [47] are increasingly recognized as essential elements of care giving. Our data suggest that at least in the population studied *these issues constitute the change in current health care most desired by the patients*. While not featuring much in older studies [48], a similar strong desire for patient autonomy was already identified by recent research [28,49-51]. To answer those needs and achieve the clinical benefits associated with better informed patients and shared decision making (Table 4), physician training programs appear to be an important tool [52]. To overcome current deficiencies these programs should be disseminated, since regardless of background or disease – being well informed by their doctors and allowed to take an active part in decisions – is a major preference for a great many patients.

## Competing interests

The author(s) declare that they have no competing interest.

## Pre-publication history

The pre-publication history for this paper can be accessed here:



## Supplementary Material

Additional File 1 Word document entitled "The form handed to the participating patients after a short verbal explanation", describing the form actually handed to the participating patients. Click here for file

## Figures and Tables

**Figure 1:**
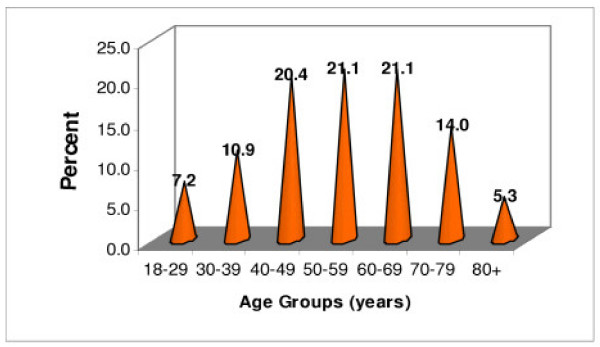
Distribution of participants by age groups (n = 274).

**Table 1:** Characteristics of 274 participating patients (in percent).

**Sex**	Male	44
	Female	56
**Origin**	Western countries	52
	Israel	27
	Arab countries	18
	Other	3
**Education**	Elementary	17.5
	High school	47.5
	University	35
**Income**	Low	36
	Medium	56
	High	8
**Location**	Hospitalized	58
	Ambulatory	42
**Main Diagnosis**	Cardiovascular	36
	Acute infection	18
	Respiratory	10
	Neurological	10
	Gastrointestinal	9
	Metabolic	4
	Other	13

**Table 2:** Patient's first and second priorities for a change in three'relational' and three 'organizational' aspects* of their current medical care (n = 274); (percent rounded to nearest 0.5).

**A) Aspects of patient-physician relationship**		
**Aspect**	**First choice**	**Second choice**

	**Number (%)**	**Number (%)**

Autonomy^#^	75 (27.5)	36 (13.0)
Continuity	39 (14.0)	54 (19.5)
Time	31 (11.0)	34 (12.5)
		
**B) Aspects of the organisation of medical care**		

**Aspect**	**First choice**	**Second choice**

	**Number (%)**	**Number (%)**

Access	49 (18.0)	54 (19.5)
Queue	45 (16.5)	62 (22.5)
Cost	35 (13.0)	34 (12.5)
		
Total	274 (100)	274 (100)

* For details please see under 'Methods' and [Additional file 1]. Both the wish for easier access and (to a lesser degree) a shorter queue, can be viewed as also pertaining to increased patient command and autonomy.

# 'Autonomy' actually refers to "Information/autonomy" as discussed in the text.

**Table 3:** The five most 'popular' patients' selections of combination of preferences for a change in their medical care*.

**Combination selected **(First → Second choice)	**Number**	**Percent**
Autonomy^# ^→ Access	21	8
Autonomy^# ^→ Continuity	19	7
Access → Queue	18	7
Autonomy^# ^→ Queue	17	6
Queue → Access	16	6
		
Total	91	34%

* Five out of 30 possible combinations were selected by >5% of the 274 patients (% rounded to the nearest full number). Full details given under 'Methods' and [**see **Additional file 1].

# 'Autonomy' actually refers to "Information/autonomy" as discussed in the text.

**Table 4:** Some advantages suggested by research of providing full information to the patient and of shared decision-making*.

▪ Better patient outcomes (5, 29).
▪ Improved patient satisfaction (4, 30).
▪ Increased patient trust (31, 32).
▪ Decreased symptom burden (4).
▪ Decreased use of resources (4).
▪ Increased provision of preventive services (33).
▪ Increased patients' knowledge about medications and management (34).
▪ Better patient adherence and compliance with treatment and medical advice (6).
▪ Improved decisions, since decisions by physicians alone may be susceptible to bias or affected by cultural differences (35, 36) and substituted judgments are often incorrect (37).
▪ Informed consent *requires *that patients be told their diagnoses, their prognoses and the risks and benefits of all proposed treatment options, including non treatment. Patient autonomy is an ethical and legal right of all patients (38).
