# Supplementary material for: Information and shared decision-making are top patients' priorities
Source: BMC Health Serv Res. 2006 Feb 28;6:21. doi: 10.1186/1472-6963-6-21 (PMC1431526; doi:10.1186/1472-6963-6-21)
Supplement: Additional File 1 — Word document entitled "The form handed to the participating patients after a short verbal explanation", describing the form actually handed to the participating patients. [file 1472-6963-6-21-S1.doc]

**Additional file 1. The form handed to the participating patients after a short verbal explanation (see under "Methods')*.**

If you could improve ONE thing in your medical care:

What would your **FIRST priority for a change** be? What would you like the *most* to be *different*?

(Please select and mark one option only)

- That the same doctor will see you each time
- That the doctor would devote more time to you
- That you will have to wait less time for tests
- That you could apply directly to specialists or to the hospital
- That you will have to pay less for the drugs
- That the doctor will explain to you all about the disease and its treatment and will let you decide together******

Male / Female

Hospitalized / Clinic

Age:

Born in (country):

Education: Elementary High-school University

Income: Low Medium High

Main problem:

What would your *second* priority for improvement be?

***** To avoid bias related to an item's place on the list, six different types of forms were prepared, each with a different order of the various choices.

****** In Hebrew, each item was not longer than 1 line.
